# Modification of m5C regulators in sarcoma can guide different immune infiltrations as well as immunotherapy

**DOI:** 10.3389/fsurg.2022.948371

**Published:** 2023-01-06

**Authors:** Shusheng Wu, Mengge Li, Rixin Su, Hao Shen, Yifu He, Yangfan Zhou

**Affiliations:** ^1^ Department of Medical Oncology, The First Affiliated Hospital of USTC, Division of Life Sciences and Medicine, University of Science and Technology of China, Hefei, China; ^2^ Department of Medical Oncology, Anhui Provincial Hospital Affiliated to Anhui Medical University, Hefei, China; ^3^ Department of Orthopedics, The First Affiliated Hospital of Anhui Medical University, Hefei, China

**Keywords:** sarcoma, m5C, tumour microenvironment, prognosis, immunotherapy

## Abstract

**Background:**

Recent studies have found that 5-methylcytosine (m5C) modulators are associated with the prognosis and treatment of cancer. However, the relevance of m5C modulators in sarcoma prognosis and the tumour microenvironment is unclear.

**Methods:**

We selected 15 m5C regulators and performed unsupervised clustering to identify m5C modification patterns and differentially expressed genes associated with the m5C phenotype in The Cancer Genome Atlas (TCGA) sarcomas. The extent of immune cell infiltration in different clustering groups was explored using single-sample gene set enrichment analysis and estimation algorithms. A principal component analysis algorithm-based m5C scoring protocol was performed to assess the m5C modification patterns of individual tumors.

**Results:**

We identified two distinct m5C modification patterns in the TCGA sarcoma cohort, which possess different clinical outcomes and biological processes. Tumour microenvironment analysis revealed two groups of immune infiltration patterns highly consistent with m5C modification patterns, classified as immune inflammatory and immune desert types. We constructed m5C scores and found that high m5C scores were closely associated with leiomyosarcoma and other subtypes, and were associated with poorer prognosis, lower PD-L1 expression, and poorer immunotherapy outcomes. The best application was validated against the m5C database.

**Conclusion:**

We constructed an m5C score for sarcoma based on the TCGA database and identified a poorer prognosis in the high m5c score group. The stability and good prognostic predictive power of the m5C score was verified by an external database. We found that sarcomas in the low m5C score group may have a better response to immunotherapy.

## Introduction

Sarcoma is characterised by soft tissue sarcoma and bone sarcoma (1). It is a heterogeneous mesenchymal malignancy that may occur at any age, and the prevalence rate of 1% in adults is significantly lower than the prevalence rate of 15% in children. Unlike other common cancers, sarcoma can occur at almost any anatomical site (1). Although previous studies have found some success with anthracyclines in rhabdomyosarcoma, Ewing's sarcoma, and osteosarcoma, most soft tissue sarcomas are resistant to conventional cytotoxic chemotherapy (2). When surgery and radiation therapy fail, there are few effective treatment options for palliation of tumour progression (3). Therefore, the development of new therapeutic targets is necessary for sarcomas.

Recently, immune checkpoint inhibitors (ICIs), including programmed cell death protein 1 (PD-1) inhibitors, have yielded promising results in sarcomas (4). In an open-label, multicentre phase II study, patients with advanced osteosarcoma were investigated. All patients were treated with pembrolizumab, a PD-1 inhibitor, in combination with cyclophosphamide, with an objective response rate (ORR) of 6.7% and 6-month progression-free survival probability of 13.3% (5). The SWONG S1609 study enrolled patients with metastatic or unresectable angiosarcoma. All patients received a combination of ipilimumab and nivolumab, and the trial results demonstrated a 25% ORR and a 38% probability of 6-month progression-free survival (6). The above clinical trials indicated the efficacy of ICIs in sarcoma; however, more than 75% of patients also failed to benefit from ICI therapy. The efficacy of ICIs involves the impact of potential cellular and molecular pathways of dynamic interactions between tumour mesenchyme, tumour cells, and immune infiltration throughout the tumour microenvironment in sarcoma tissue (7). In addition, progression in sarcoma therapy relies on biomarkers with prognostic and predictive value to select patients most likely to benefit from ICIs and serve as effective therapeutic targets. The understanding of the tumour microenvironment (TME) has also provided a better understanding of the critical role of the microenvironment in which tumour cells grow and survive in tumour development. In addition to cancer cells, the TME contains various immune cells, mesenchymal cells, and various cytokines (8).

5-methylcytosine (m5C) has potential as a new epigenetic marker that may play a key role in RNA and DNA modifications and cellular metabolic processes (9). Emerging evidence has demonstrated that m5C is closely associated with the development of malignant diseases (10, 11). For example, high expression of NSUN5 promotes cell proliferation by regulating the cell cycle of colon cancer cells (12). The methylation regulator DNMT1 is hypermethylated and overexpressed in pancreatic cancer, which can be initiated by miR-148a and lead to proliferation and invasion of pancreatic cancer cells (13). Regulatory factors of m5C RNA methylation can predict the clinical prognostic risk of triple-negative breast cancer patients and affect the tumour immune microenvironment (14). Risk modelling of m5C-associated lncRNAs may be a promising prognostic tool for lung adenocarcinoma (15). However, the role of m5C modulators in sarcomas is poorly understood. Wang et al. (16) used 255 soft tissue sarcoma samples in TCGA involving six m5C regulatory genes to analyze the correlation between copy number variation and soft tissue sarcoma prognosis. The results suggested that YBX1-based copy number loss was associated with poorer OS. However, the study did not construct a scoring model for m5C. Therefore, a comprehensive exploration of multiple m5C regulator-mediated TME cell infiltration properties will contribute to a deeper understanding of TME immune regulation.

In the present study, we comprehensively evaluated the influence of m5C on TME in sarcoma patients using the Cancer Genome Atlas (TCGA) and Gene Expression Omnibus (GEO) datasets. Based on unsupervised clustering analysis, we identified two different m5C modification patterns and revealed that the TME was completely different between the two groups. Finally, we constructed an m5C scoring signature to score individual tumours and assess the m5C modification pattern in individual patients. Based on the m5C scoring signature, we predicted the usefulness of this signature for assessing the efficacy of immunotherapy. This study demonstrates that m5C modification has positive implications for the clinical prognosis and treatment of patients with sarcoma.

## Materials and methods

### Data acquisition for sarcoma

The 256 sarcoma samples from TCGA (www.cancer.gov) containing the gene expression matrix and clinical information were used as the training set. The validation set data, which includes GSE17674, GSE63157, and GSE30929, were obtained from GEO (http://www.ncbi.nlm.nih.gov/geo) (17–19). GSE17674 and GSE63157 are Ewing's sarcoma datasets containing complete overall survival (OS) information. GSE63457, a liposarcoma dataset containing complete disease-free survival information for 140 patients, consists of two subsets, GSE63155 and GSE63156. We merged these three datasets and used the R software (https://www.r-project.org/, version 3.5.3) package “sva” (20) to correct for batch effects (Supplementary Figure S1A). In addition, the TCGA sarcoma cohort was used for copy number variation (CNV) analysis. Data were analysed using the R “Bioconductor” package.

### Unsupervised clustering of 15 m5C-related regulators

We selected 15 m5C regulators from the literature (21), including “NSUN5, ALYREF, DNMT3B, YBX1, NOP2, NSUN2, TET3, TET2, NSUN6, DNMT3A, NSUN3, DNMT1, NSUN4, TRDMT1, and NSUN7”. The DNMT family, NSUN family, NOP2, and DNMT3A were “writers”; TET2 and TET3 were “erasers”; and YBX1 and ALYREF were “readers”. We applied the R package “ConsensuClusterPlus” to perform an unsupervised cluster analysis to group sarcoma patients according to the expression of 15 m5C regulators (22). Survival curves and 3D principal component analysis (PCA) plots were used to verify the validity of clustering.

### Gene set enrichment analysis

To explore the enrichment differences between clustering groups, we obtained the Kyoto Encyclopedia of Genes and Genomes (KEGG) from the online website gene set enrichment analysis (GSEA) (http://www.gsea-msigdb.org/) pathway gene set (c2.cp.kegg.v7.2.symbols) and Hallmarker gene set (h.all.v7.4.symbols). We used the R package “ClusterProfiler” to perform GSEA with a set cut-off value of *p*-value < 0.05.

### Differences in immune cell infiltration between clustered subgroups

The single-sample GSEA (ssGSEA) algorithm was used to quantify the degree of enrichment of immune cells in each sample and was performed using the R package “GSVA” (23). The gene set of 29 immune cells was obtained from the literature (24). Box plots were used to visualise the differences in immune cell abundance between the two subgroups.

### Identification and clustering of prognosis-related differentially expressed genes among different phenotypes of m5C

To define m5C-related genes, we divided the samples into two clusters based on the expression of m5C regulators. The R package “limma” was used to identify differentially expressed genes (DEGs) between two clusters (25), with an adjusted *p*-value < 0.001 as the filtering criterion. Univariate Cox regression analysis was used to identify the DEGs that were related to prognosis (*p* < 0.05). Similarly, we used unsupervised clustering methods to perform clustering analysis of prognosis-related DEGs among different phenotypes of m5C and explore in depth the biological differences between different clustering groups.

### Construction of the m5C score

To quantify the m5C modification pattern of individual tumours, we constructed an m5C scoring system to assess the m5C modification pattern of individual sarcoma patients. Based on the m5C prognosis-related differential genes we obtained above, we performed a PCA approach to construct an m5C-related gene signature. Both PCA1 and PCA2 were selected as signature scores. A similar approach from previous studies to define m5C score (26, 27):

m5Cscore=∑(PC1i+PC2i)

where I is the expression of m5C phenotype-related genes.

### Correlation of m5C score with other biological information

In the literature, we downloaded a gene set used to store genes associated with certain biological processes (28, 29) (Supplementary Table S1). The correlation of the m5C score with each biologically relevant pathway was calculated by R package “corrplo” and a correlation heat map was drawn to visualise the correlation.

### Correlation of m5C score with immune microenvironment and survival information

Using the optimal cut-off values, we obtained high-risk and low-risk groups based on m5C scores. We calculated the immune score of sarcoma patients using the R package “ESTIMAT” (30). The difference in immune infiltration between the two groups can be clearly seen by comparing the differences in immune scores between the high-risk and low-risk groups. Differences in immunotherapy between the two groups, PD-L1 and HLA families, were included in the study. Additionally, we downloaded GSE78220, a dataset consisting of 27 patients with melanoma treated with a PD-1 inhibitor. Based on our constructed m5C score signature, we assessed whether this m5C modification signal could predict patient response to ICIs. Finally, we validated the model with the same m5C score model in the GSE17674, GES63157, and GSE30926 cohorts to assess the predictive power of the model for survival of sarcoma patients. A final prognostic assessment model was constructed using Cox regression analysis of clinical factors in the TCGA cohort.

### Statistical analysis

Correlation between m5C-related regulators and the correlation between m5C score and biologically relevant pathways were based on Pearson correlation coefficients. Comparisons between the two groups in this study were based on the Wilcoxon test. We used the Kaplan–Meier method to plot survival curves for the prognostic analysis, and log-rank tests were used to determine the significance of differences. Differences were considered statistically significant at *p* < 0.05.

## Results

### Landscape of genetic variation of m5C regulators in sarcoma

**Figure 1:**
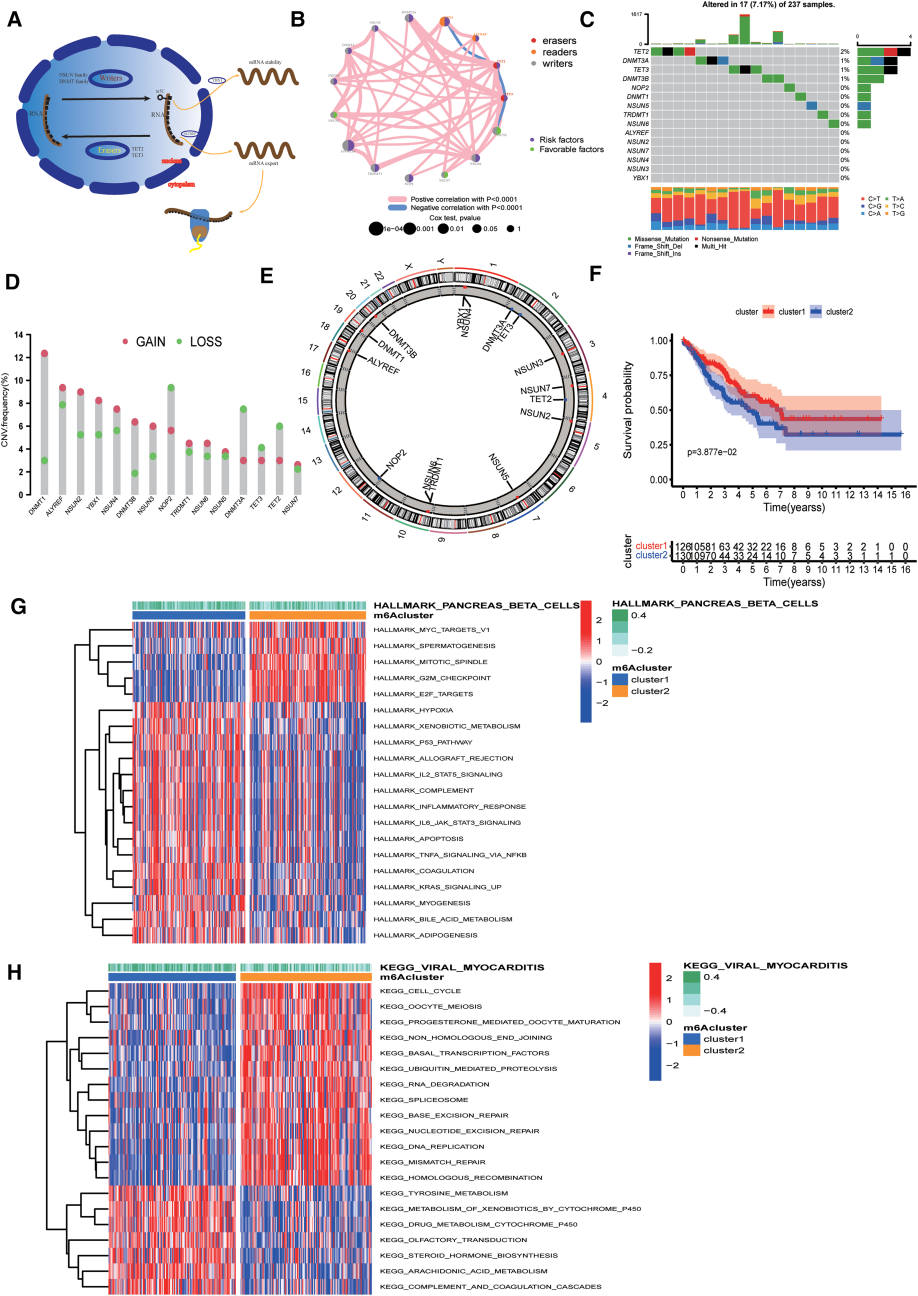
Landscape of genetic alterations of 5-methylcytosine (m5C) regulators in sarcoma. (**A**) Biological functions of m5C in RNA metabolism. (**B**) Interaction of 15 m5C regulators. The color represents the type of m5c. The size of the circle indicates the prognostic effect of the *p*-value assessment. The color inside the circle represents the prognostic effect, including protective factors (green) and risk factors (purple). (**C**) Mutation frequencies of 15 m5C regulators in 256 TCGA sarcoma samples. (**D**) Copy number variation (CNV) mutation frequencies of the 15 m5C regulators. (**E**) Location of CNV alterations of m5C regulators on chromosomes. (**F**) Survival curves showing a poor prognosis for cluster2. (**G**) Results of Hallmarker genomic enrichment analysis. (**H**) Results of KEGG set enrichment analysis.

In this study, we selected 15 m5C regulators and elucidated the dynamic regulatory mechanism of m5C regulators in cells in the model map (Figure 1A). By analysing the correlation between the 15 m5C regulators, we discovered that most m5C regulators showed a significant positive correlation with each other (Supplementary Figure S1B). Univariate Cox regression demonstrated that m5C was a risk factor for patient survival (Supplementary Figure S1C). The PPI protein interaction network map obtained from the “STRING” online website also visualises the complex interactions between m5C regulators (Supplementary Figure S1D). Therefore, we hypothesised that the causative factor of sarcoma may be related to the overexpression of m5C regulators. A comprehensive illustration of the interactions of 15 m5C regulators and their prognostic significance in sarcoma patients is illustrated in the m5C regulator network (Figure 1B). We determined the incidence of somatic mutations in sarcomas using 15 m5C regulators. Among the 257 samples, a total of 19 cases (8.02%) had genetic alterations in m5C regulators, and the highest mutation frequency was observed in TET2 (Figure 1C). CNV alteration frequency showed that CNV alteration was more common in 15 m5C regulators, and most of them were concentrated in copy number amplification, while the frequency of CNV deletion was higher in NOP2, DNMT3A, TET2, andTET3 (Figure 1D). The location of CNV alterations in m5C regulators on chromosomes is shown in Figure 1E.

### Identification of m5C methylation modification patterns mediated by 15 regulators

Based on the expression of 15 m5C regulators, we divided the samples into two groups, namely cluster1 and cluster2, using unsupervised clustering (Supplementary Figure S2A). Cluster1 had 126 samples and cluster2 had 130 samples. Survival curves suggested that cluster2 had a poorer prognosis (Figure 1F).

### Biological differences between clusters of m5C regulators

**Figure 2:**
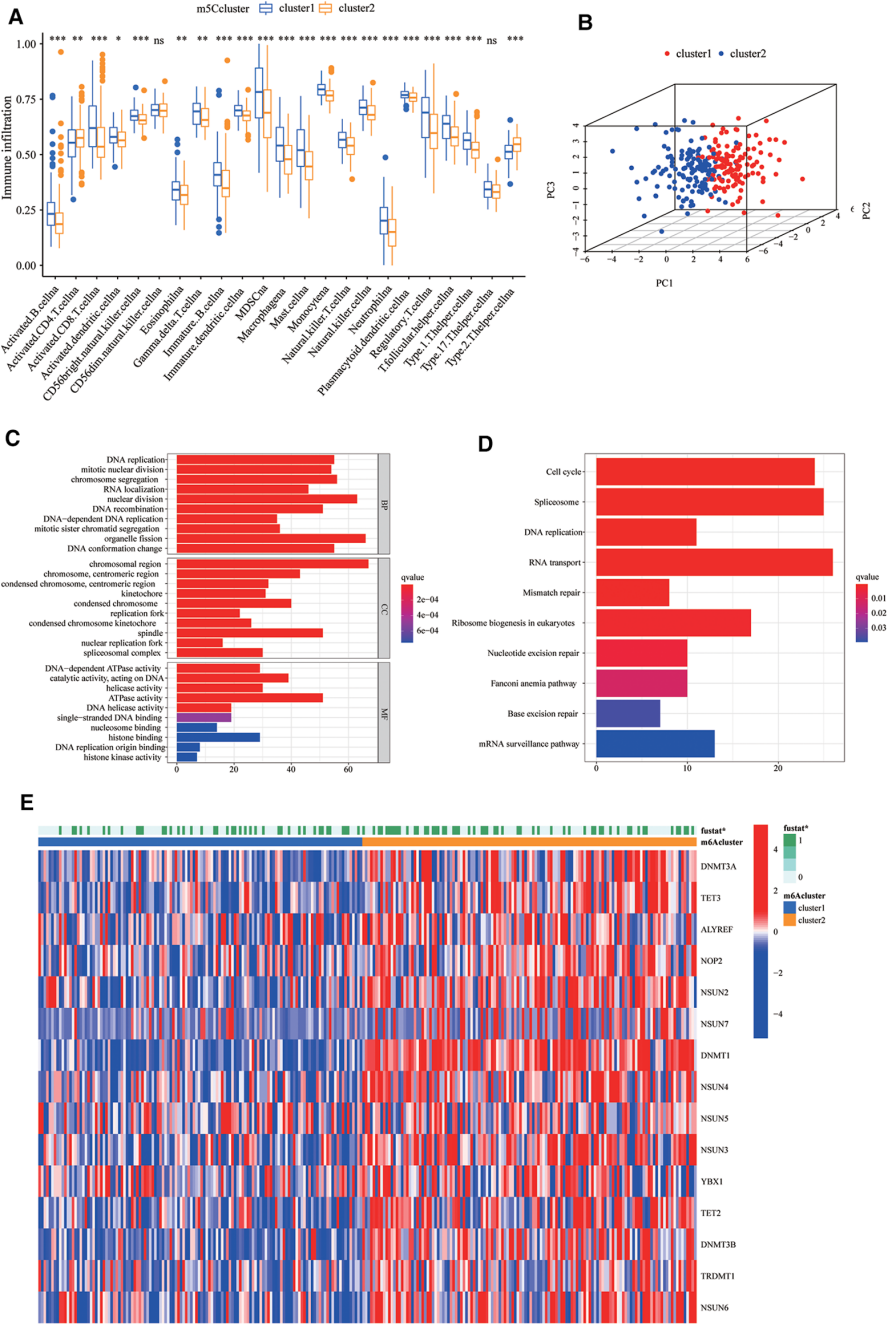
Biological differences and differentially expressed genes (DEGs) between clusters of m5C regulators. (**A**) The differences in the enrichment of 28 immune cell species between the two groups. The boxplot extends from the 25th percentile to the 75th percentile, with the center parallel line indicating the median. (**B**) Principal component analysis (PCA) plots demonstrate that the 5-methylcytosine (m5C) modification patterns are significantly different between the two clusters. (**C,D**) Gene ontology (GO) and Kyoto Encyclopedia of Genes and Genomes (KEGG) pathway enrichment analysis was used to explore the biological information of m5C phenotype-related differentially expressed genes (DEGs). (**E**) The different expression of m5C regulators in two clusters.

To explore the biological differences between the two groups, we performed ssGSEA enrichment analysis on the Hallmarker and KEGG pathway gene sets. The results demonstrated that cluster2 was associated with tumour proliferation, oncogenic activity, cell cycle, and RNA degradation, while cluster1 was highly enriched in immune regulation as well as metabolic pathways (Figures 1G,H). The results of immune cell infiltration in both groups showed that most immune cells were highly enriched in cluster1 (Figure 2A). The PCA plot also indicated a significant difference between the two clusters (Figure 2B). Taken together, our study suggests that there are significant differences between the two m5C modification patterns in terms of immune microenvironment, with cluster1 being the immune inflammatory type with a better prognosis and cluster2 being the immune desert type with a relatively poor prognosis. The m5C regulatory factor expression was significantly higher in cluster2.

### M5c phenotype-related DEGs in sarcoma

**Figure 3:**
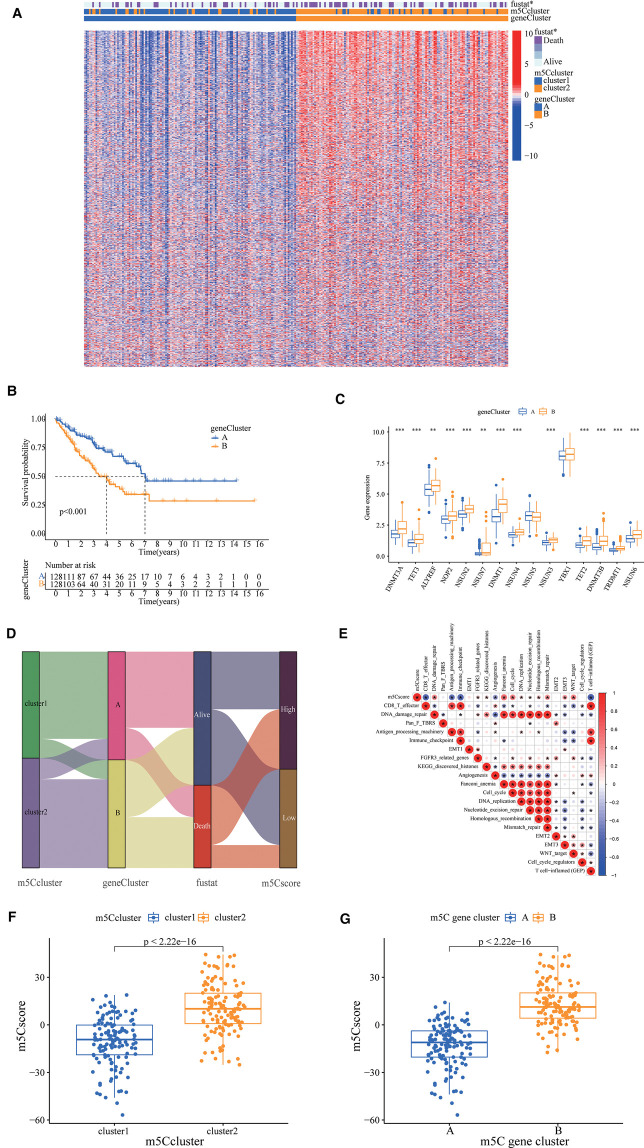
5-methylcytosine (m5C) phenotype-related differentially expressed genes (DEGs) in sarcoma. (**A**) A differential expression heat map was drawn to visually illustrate the differences in expression of 990 m5C phenotype-related DEGs between the two groups. (**B**) The survival curve showed different survival rate between two groups. (**C**) The prominent differences in the expression of m5C regulators, the expression of m5C regulators in cluster B was significantly higher than cluster A. (**D**) A Sankey plot to show the correlation of m5C score, survival status, m5C cluster, and gene cluster. (**E**) Correlations between m5C score and the known gene signatures in The Cancer Genome Atlas cohort using Pearson analysis. Negative correlation was marked with blue and positive correlation with red. (**F**) Wilcoxon test showed a significant difference in the m5C score between m5C gene clusters, and the score of Cluster A was significantly lower than that of Cluster B. (**G**) The m5C score of cluster1 in the m5C cluster is also significantly lower than that of cluster2.

To investigate the potential biological functions of the two m5C modification patterns, we used the R package “limma” to obtain the differential genes between cluster1 and cluster2. Genes closely associated with survival were then screened using univariate Cox regression analysis (Supplementary Figure S2B, Supplementary Table S2). We identified 990 DEGs associated with the m5C phenotype and performed gene ontology (GO) and KEGG enrichment analyses. The histogram revealed that these genes were significantly enriched in biological processes, such as RNA modification, DNA modification, transcription, and cell proliferation (Figures 2C,D). These m5C phenotype-related DEGs were further demonstrated to be closely related to m5C modifications. There was also a significant difference in the expression of M5C regulators between the two clusters (Figure 2E). Similarly, we used an unsupervised clustering approach to divide the samples into two stable groups (cluster A and cluster B) based on the expression of 990 m5C phenotype-related DEGs (Supplementary Figure S2C). The differential expression heatmap was drawn to visually illustrate the differences in the expression of 990 m5C phenotype-related DEGs between the two groups (Figure 3A). We found significant upregulation of m5C phenotype-related DEG expression in cluster B with a significantly worse prognosis (Figure 3B). In the two m5C gene clusters, prominent differences in the expression of m5C regulators were observed, and the expression of m5C regulators in cluster B was significantly higher than that in cluster A (Figure 3C). This was consistent with the expected results for m5C methylation modification patterns.

### Immune regulation differences in m5C-related phenotypic genes

To reveal the role of m5C-related phenotypic genes in the regulation of TME immunity, we investigated the expression of chemokines and cytokines in two gene clusters. Cytokine and chemokine transcripts were obtained from published literature (8), and the transcripts of the transforming growth factor (TGF)β/EMT pathway are SMAD9, Twist1, CLDN3, TGFBR2, ACTA2, COL4A1, ZEB1, and VIM. Transcripts of immune checkpoints are PD-L1, CTLA-4, IDO1, LAG3, HAVCR2, PD-1, PD-L2, CD80, CD86, TIGIT, and TNFRSF9. Immune-activated transcripts were TNF, IFNG, TBX2, GZMB, CD8A, PRF1, GZMA, CXCL9, and CXCL10. We found that mRNAs associated with the TGFβ/EMT pathway were partially upregulated in cluster B (Supplementary Figure S2D). In contrast, cluster A was significantly highly expressed in immune checkpoints as well as in immune activation transcript-associated mRNAs (Supplementary Figure S2E,F).

### Construction of the m5C score

The optimal cut-off value for the m5C score was set based on the m5C score and the patient's survival information, and the sample was divided into high and low scoring groups. There were 139 and 117 samples from the high and low subgroups, respectively. Considering the complexity of the quantification of m5C modification, we plotted a Sankey plot to show the correlation of m5C score, survival status, m5C cluster, and gene cluster (Figure 3D). The heatmap of the correlation between m5C score and biologically relevant features indicated a significant negative correlation between m5C score and immune activation process and a positive correlation with mesenchymal-related features and cell proliferation-related features (Figure 3E). The m5C score of cluster1 was significantly lower than that of cluster2 (Figure 3F). In addition, the m5C score of cluster A in the m5C cluster was also significantly lower than that of cluster B (Figure 3G).

**Figure 4:**
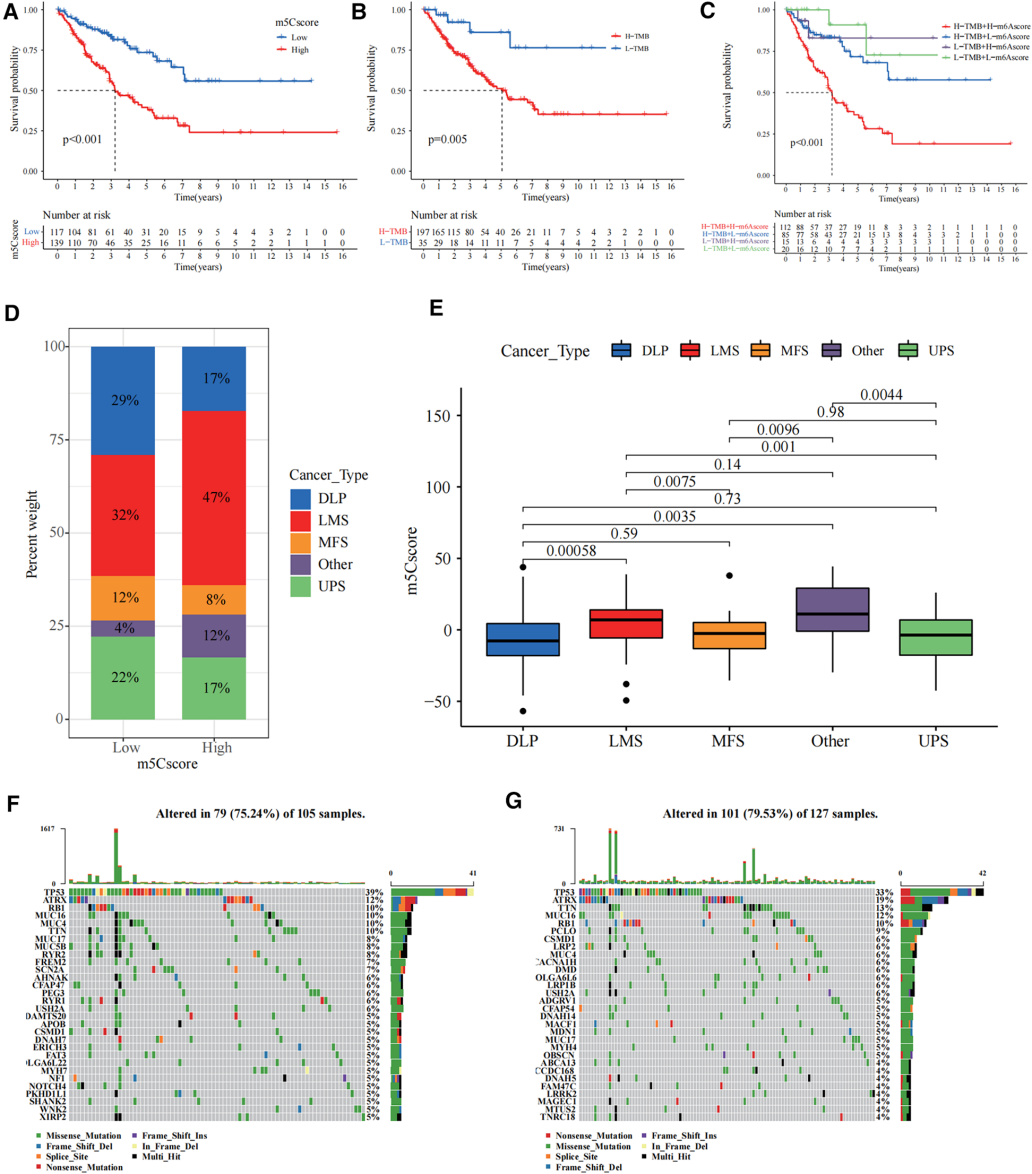
Construction of the 5-methylcytosine (m5C) score and relevance of its clinical features. (**A**) The survival curve showed that the high m5C score group had a worse prognosis (*p* < 0.001). (**B**) The survival curve showed that the high tumour mutation burden (TMB) score group had a worse prognosis (*p* = 0.005). (**C**) Combining the m5C score and TMB score, we found that the group with high m5C and TMB scores had the worst prognosis. (**D**) The bar chart shows the percentage distribution of different sarcoma subtypes in the high and low m5C scoring subgroups. (**E**) Distribution of the m5C score in the different sarcoma subtypes. The differences between every two groups were compared through the Kruskal–Wallis H test. (**F,G**) The waterfall plots illustrate the differences in somatic mutations between the high (right) and low (left) m5C score groups.

Survival curves showed that the high m5c subgroup had poorer prognosis (Figure 4A). According to the tumour mutation burden (TMB) score, we divided the patients into high and low TMB groups, and we found that patients in the high TMB group had a significantly worse prognosis (Figure 4B). Combining the m5C and TMB scores, we found that the group with high m5C and TMB scores had the worst prognosis (Figure 4C). Sarcomas in TCGA are classified as dedifferentiated liposarcoma (DLP), smooth muscle sarcoma (LMS), undifferentiated pleomorphic sarcoma (UPS), mucinous fibrous sarcoma (MFS), and others. We also investigated the relationship between the m5C score and sarcoma subtypes and found that LMS and others were significantly enriched in the high m5C score group, while the other three sarcomas DLP, MFS, and UPS had relatively low m5C scores (Figures 4D,E). Finally, we plotted waterfall plots to visually illustrate the differences in somatic mutations between the high and low m5C score groups, which showed no significant difference in somatic mutation rates between the two groups, with the low score group being 75.24% and the high score group being 79.53% (Figures 4F,G).

### Guidance of m5C score for immunotherapy

**Figure 5:**
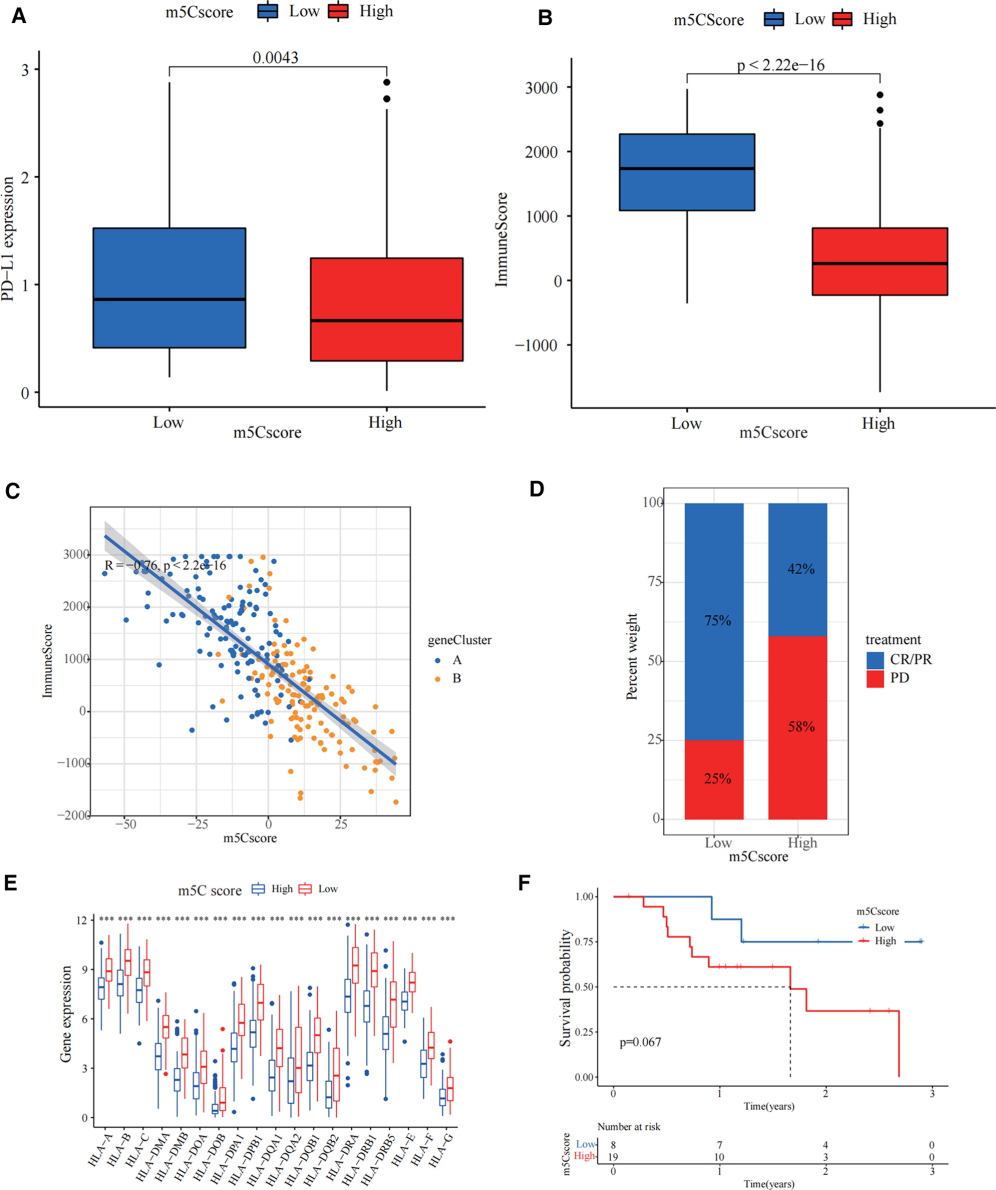
Guidance of 5-methylcytosine (m5C) score for immunotherapy. (**A**) PD-L1 expression was significantly higher in the low m5C score group than in the high m5C score group (*p* = 0.0043). (**B**) The immune score in low m5C score group is higher than high m5C score group (*p* < 2.22 × 10^−16^). (**C**) Immune scoring also showed a significant negative correlation with m5C score. (**D**) The bar chart demonstrates the different immunotherapy effects in high and low m5C score groups. (**E**) HLA family expression was significantly higher in the low m5C score group than in the high m5C score group (***p < 0.001). (**F**) Kaplan-Meier curves for high and low m5C score patient groups in the GSE78220 cohort (Log-rank test *p* = 0.067).

We investigated the significance of m5C for guiding immunotherapy in sarcoma patients and found that PD-L1 expression was significantly higher in the low m5C score group than in the high m5C score group through the study of PD-L1 expression (Figure 5A). Immune scoring of the low m5C score group was significantly higher than that of the high m5C score group (Figure 5B). Immune scoring also showed a significant negative correlation with m5C scores (*R* = −0.76, *p* < 2.2 × 10^−16^, Figure 5C). In summary, the low m5C score group may have a better response to immunotherapy. To test our hypothesis, based on the GSE78220 melanoma immunotherapy cohort (31), we investigated whether m5C modification signalling could predict patient response to ICIs. Compared to patients with high m5C scores, the proportion of patients with low m5C scores who received anti-PD-1 inhibitor with partial response (PR) and complete response (CR) was significantly higher (75%) than patients with progressive disease (PD) (25%). The opposite was true for patients with a high m5C score (Figure 5D). HLA family expression was significantly higher in the low m5C score group than in the high m5C score group (Figure 5E). Survival curves can be found for patients with low m5C scores, showing good survival rates (Figure 5F). This demonstrates that patients with low m5C have a more pronounced immunotherapeutic advantage.

### Validation of m5C modification patterns and clinical application

**Figure 6:**
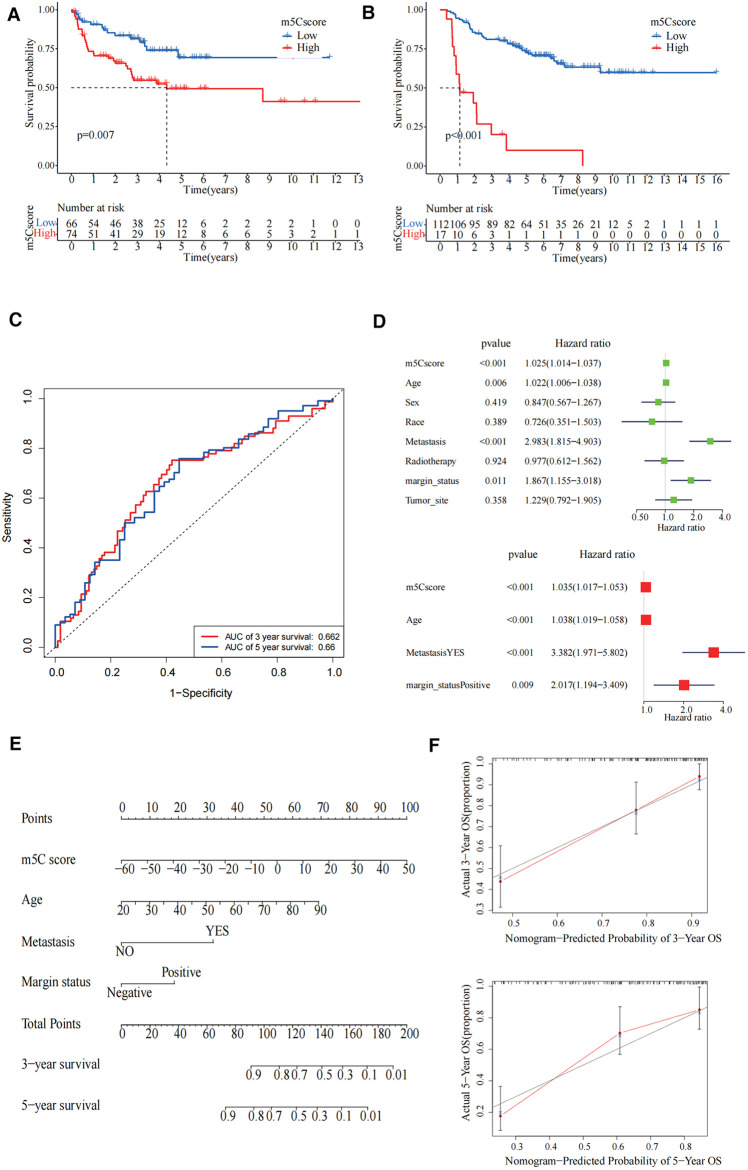
Validation of 5-methylcytosine (m5C) modification patterns and clinical application. (**A**) The survival curve showed high m5C score group had worse prognosis in GSE30926 cohort (*p* = 0.007). (**B**) The survival curve showed that the high m5C score group had a worse prognosis in the GSE17674 and GES63157 cohorts (*p* < 0.001). (**C**) The predictive value of the m5C score in The Cancer Genome Atlas (TCGA)-sarcoma cohorts. Three-year area under the curve (AUC) = 0.662, 5-year AUC = 0.66. (**D**) Univariate and multivariate Cox regression analysis for m5C score in TCGA cohort shown by the forest plot. (**E**) Nomogram for predicting patient survival at 3-year and 5-year. (**F**) Calibration curve showed the prediction power of nomogram in 3 years and 5 years.

To further examine the stability of the m5C scoring signature, we applied the m5C scoring signature and formulae built in the TCGA cohort to the GSE17674, GES63157, and GSE30926 cohorts. The survival curves demonstrated that our constructed signatures had a good predictive power for survival (Figures 6A,B). The area under the curve values of the receiver operating characteristic curves in TCGA validated the predictive ability of our model (Figure 6C). In univariate Cox regression analysis, m5C score, age, metastasis, and margin status were significantly correlated with survival, and these four items remained significant in the multivariate Cox regression analysis (Figure 6D). For the convenience of clinicians, we constructed a clinical prognostic nomogram using these four factors, and the calibration curves at 3 and 5 years also demonstrated the good prognostic predictive ability of our nomogram (Figures 6E,F).

## Discussion

Previous studies have shown that frequently mutated genes such as PIK3CA and TP53 are associated with the prognosis of sarcoma (32). In addition, epigenetic regulation plays a key role in the tumorigenesis of sarcoma (33). Research has shown that various RNA modifications are closely associated with various diseases, with N6-methyladenosine, m5C, N1-methyladenosine (m1A), and pseudouridine being the most widespread modifications of RNA (10). In a recently research study, researchers constructed the Regulator-Related Score using 32 RNA-modifying regulators from the TCGA soft tissue sarcoma cohort and found poor prognosis for the low scoring group (34). Wu et al. (35) constructed a prognostic model of long non-coding RNAs associated with m1A/m5C/m6A in osteosarcoma associated with patient OS based on the TARGET database. Results revealed that a risk signature based on two m1A/m5C/m6A-associated lncRNAs could be predictive of prognosis and immune landscape in osteosarcoma patients. However, there are no studies to understand the immune microenvironment of sarcoma based on the m5C score. In the present study, we focused on exploring m5C modification patterns in the context of the immune microenvironment of sarcomas. We clarified the role of different m5C modification patterns in TME cell infiltration, and deepened our understanding of the anti-tumour immune response to TME to guide more effective immunotherapeutic strategies.

Based on the analysis of m5C regulator mutations in the TCGA cohort of sarcomas, we found that TET2 had the highest mutation rate. Previous studies have found that TET2 mutations are an extremely common type of mutation in leukaemia. TET2 recovery reverses the self-renewal of abnormal haematopoietic stem and progenitor cells *in vitro* and *in vivo* and inhibits leukaemia progression (36). In this study, based on the expression of m5C regulators, we identified two different m5C modification patterns. They suggest different prognoses and tumour immune microenvironments, with cluster1 having a better prognosis and the expression of m5C regulators being relatively lower. The results of the enrichment analysis show that cluster1 was characterised by an enrichment of immune cells as well as immune-related biological functions. In contrast, cluster2, with survival rate and the degree of immune infiltration much lower than those of cluster1, was characterised by biological functions related to cell proliferation and significantly enriched tumour activity. This is consistent with past studies suggesting that poor prognosis in sarcoma patients is often closely related to tumour proliferation and metastasis (37). The composition of immune cells also differs between the different patterns of RNA-modified regulators (34). In addition, the differential genes identified from different m5C modification patterns, which we found to be closely associated with RNA vs. DNA modifications, suggest that these genes can be considered as m5C phenotype-associated genes. Based on the clustering typing of m5C phenotype-associated genes, we obtained similar results to the clustering typing of m5C regulators, where cluster B had a poorer prognosis and m5C phenotype-associated gene expression was significantly higher. Several genes associated with stromal activation had elevated expression in cluster B, while immune checkpoint and immune activation-related genes had decreased expression, again demonstrating the importance of m5C modifications in shaping different TMEs.

To better understand the heterogeneity among individual patients, we constructed the m5C score to assess the m5C modification pattern of individual tumours. The m5C score was significantly higher in tumour patients with predominantly immune rejection phenotype, and the expression of PD-L1, HLA family, and immune score was significantly negatively correlated with the m5C score, indicating that the m5C score has a significant suggestive effect on the direction of immunotherapy for patients. Our results and previous studies suggest that m5C RNA methylation regulators have the potential to become new biomarkers and therapeutic targets for various tumors (38). By clarifying the tumor microenvironment and the ability of immunotherapy to predict biomarkers in sarcoma, immunotherapy outcomes in sarcoma may be improved (39). The dataset based on PD-1 treatment validated our hypothesis. In terms of sarcoma subtypes, the m5C scores of LMS and others were significantly lower than that of other subtypes, suggesting that the efficacy of immunotherapy against LMS and others may be relatively poor and that they are better suited to target tumour proliferation and activity-related pathways, such as cell cycle and Wnt target.

To be more suitable for clinical treatment and use, we explored the association between the m5C score and clinical prognosis by combining clinical information and assessing patients' immunophenotypes, tissue subtypes, molecular subtypes, and genetic variants by m5C score to provide the most appropriate treatment plan. In addition, the m5C score can be used as an independent predictor of patient prognosis; therefore, we developed a novel prognostic nomogram to visually predict the prognosis of patients at 3 and 5 years. The m5C score can predict the efficacy of adjuvant chemotherapy and the clinical response of patients to immunotherapy. Our findings could provide new ideas for the future personalised treatment of patients with sarcoma and immune-targeted therapy.

## Conclusion

In the present study, a comprehensive and systematic analysis of m5C regulators of sarcoma was performed. A scoring model constructed based on m5C regulators can predict prognosis and guide immunotherapy. Assessment of individual m5C modification patterns may provide more effective therapeutic strategies for immunotherapy of sarcoma patients in the future.

## Data Availability

Publicly available datasets were analyzed in this study. This data can be found here: http://www.ncbi.nlm.nih.gov/geo and https://portal.gdc.cancer.gov/.
