# Supplementary material for: Modification of m5C regulators in sarcoma can guide different immune infiltrations as well as immunotherapy
Source: Front Surg. 2023 Jan 6;9:948371. doi: 10.3389/fsurg.2022.948371 (PMC9853431; doi:10.3389/fsurg.2022.948371)
Supplement: Supplementary file 3 [file Datasheet1.docx]

Supplementary Material

**
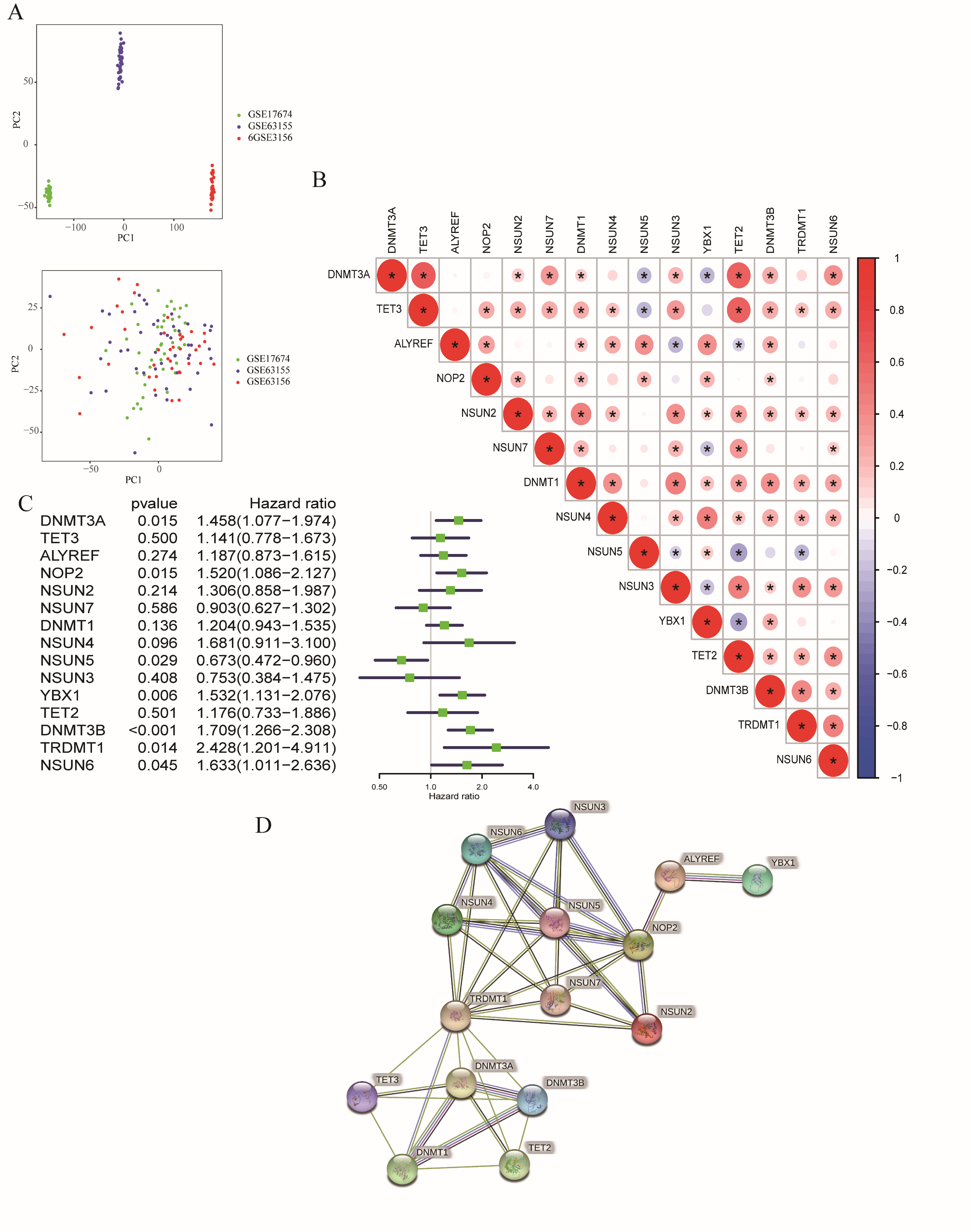
**

**Figure S1:** Correct for batch effects and correlation, univariate Cox regression and protein interaction network analysis between 15 m5C regulators. A. Correction of batch effects. B. Correlation analysis between 15 m5C regulators. C. Univariate Cox regression survival analysis of 15 m5C. D. Analysis of the protein interaction network between 15 m5C regulators.

**
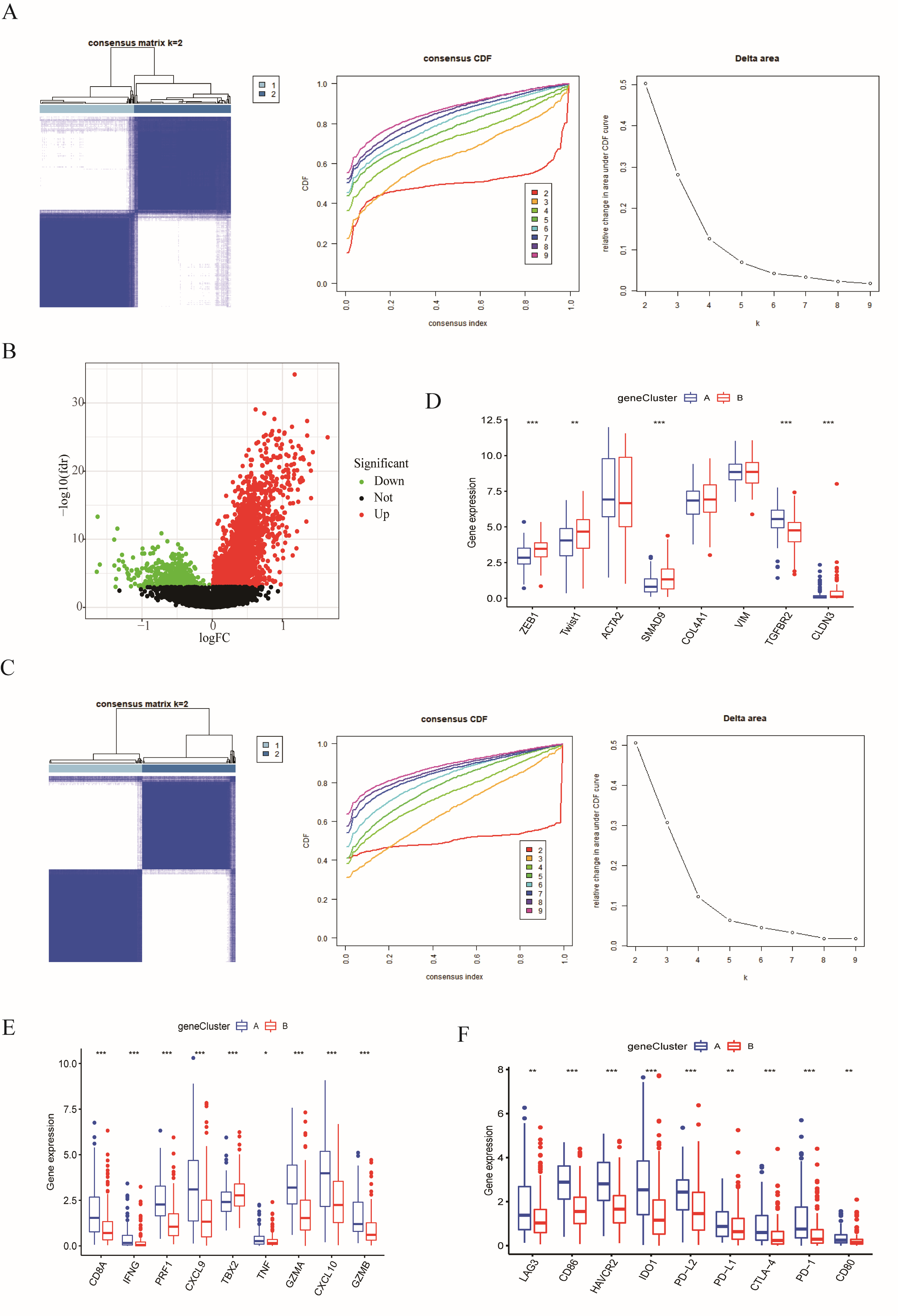
**

**Figure S2:** Sample grouping. A.The unsupervised clustering method was used to divide the samples into two groups, cluster1 and cluster2. B.The Volcano: univariate Cox regression screening for genes strongly associated with survival. C. Based on 990 DEGs associated with the m5C phenotype, the samples were divided into two stable groups (cluster A and cluster B) using unsupervised clustering. D, E, F. Immune regulation differences in m5C-related phenotypic genes in cluster A and cluster B.

**Table S1:** Storage of gene sets associated with certain biological processes.

**Table S2:** List of genes closely associated with survival screened by univariate Cox regression.
